# Seven-Channel Polyethersulfone Hollow-Fiber Membrane Preparation with Vapor-Induced Phase Separation

**DOI:** 10.3390/membranes15060175

**Published:** 2025-06-10

**Authors:** Xiaoyao Wang, Zhiyuan Hao, Rui Huang, Yajing Huang, Huiqun Zhang, Xiujuan Hao

**Affiliations:** 1 Guangdong Water Co., Ltd., Shenzhen 518021, China; 2 School of Environment, Harbin Institute of Technology, Harbin 150090, China; 3 School of Environment, South China Normal University, Guangzhou 510006, China; 4 Inner Mongolia Key Laboratory of Green Construction and Intelligent Operation and Maintenance of Civil Engineering, Hohhot 010051, China

**Keywords:** hollow-fiber membrane, polyethersulfone, vapor-induced phase separation

## Abstract

Polyethersulfone (PES) has been widely used to fabricate hollow-fiber ultrafiltration membranes due to its good oxidative, thermal, and hydrolytic stability. Typical PES hollow-fiber membranes with a single bore have limited strength and may break under uneven pressure and vibration during membrane backwashing. Multi-channel hollow-fiber membranes have stronger breaking force due to their larger cross-sectional area, but fabricating them remains challenging due to the difficulty in controlling the phase inversion process. This study uses the vapor-induced phase separation (VIPS) method to fabricate a seven-channel PES hollow-fiber membrane, and the air gap and air relative humidity can help in membrane morphology control. Moreover, carboxylic graphene quantum dots (CGQDs) are first used in ultrafiltration membranes to increase membrane porosity and hydrophilicity. We found that the membrane prepared with a 7.5% CGQD mass fraction, a 10 cm air gap, and 99% relative humidity had the highest flux and porosity; the membrane pore size distribution was concentrated at 72 nm, and the pure water flux could reach 464 L·m^−2^ h^−1^·bar^−1^. In the long-term filtration performance test, the membrane can reject more than about 15% TOC and 84% turbidity at 50 L·m^−2^ h^−1^ flux, confirming its stability for water purification applications.

## 1. Introduction

Ultrafiltration membranes typically have pore sizes between 2 and 100 nm, which can effectively remove macromolecular substances such as suspended solids, colloids, microparticles, bacteria, and viruses from water [1,2]. It is an indispensable material in advanced drinking water treatment, municipal sewage treatment, and seawater desalination pre-treatment [3,4,5]. Ultrafiltration membranes can be fabricated from polymers and ceramics; most water treatment ultrafiltration membranes are polymer-based due to their low cost and ease of manufacturing [6]. Polymer ultrafiltration membranes generally come in flat-sheet and hollow-fiber configurations. The hollow-fiber configuration provides a larger effective membrane area per unit volume of membrane module (up to 10,000 m^2^/m^3^), resulting in a reduced occupied area of membrane facilities compared to flat-sheet membranes. Additionally, the hollow-fiber membrane has a simple preparation method and self-supporting characteristics, which allow for easy production and packaging [7]. Polyethersulfone (PES) has been widely used to fabricate hollow-fiber membranes due to its good oxidative, thermal, and hydrolytic stability, as well as its mechanical properties [8]. Typical PES hollow-fiber membranes have a single bore with a diameter of 0.8–1.5 mm [1]. The small cross-sectional area results in low membrane strength, and low-strength hollow fibers can easily break or become damaged due to the uneven pressure and the vibration during membrane backwashing [9,10,11,12]. For drinking water facilities, the breaking fiber will raise the possibility of pathogens in the potable water supply and increase disinfection expenses in drinking water facilities [12]. Multi-channel hollow-fiber membranes have a higher maximum load due to a larger cross-sectional area [3,10,11]. However, preparing multi-channel hollow-fiber membranes remains challenging due to the difficulty in controlling the phase inversion process [10]. Since the cross-section of multi-channel hollow fibers is more irregular, the mass transport between solvent and non-solvent becomes difficult during the phase inversion process; macrovoids may form under instantaneous demixing when the polymer solution enters the non-solvent [13].

This study used the vapor-induced phase separation (VIPS) method to fabricate a seven-channel hollow-fiber membrane for drinking water purification. In the VIPS method, a nascent hollow-fiber membrane can cross an air gap above the coagulation bath, leading to better control of the membrane structure and more porous outer surfaces [13,14,15]. Furthermore, we first used carboxylic graphene quantum dots (CGQDs) as an additive in hollow-fiber membrane fabrication. The carboxyl groups of CGQDs can form Lewis acid–base complexes with solvents like dimethylacetamide (DMAc) and serve as a pore former by decreasing the thermodynamic stability. This can also delay phase separation by increasing the solution viscosity, so that it consists of fewer macrovoids [13,16,17]. At the same time, CGQDs can remain inside the membrane, increasing the hydrophilicity of the membrane surface, resulting in a better anti-pollution ability. Based on the above ideas, this study investigated the effects of CGQD concentrations, air gap lengths, and relative humidity on membrane structure and performance. Under optimal preparation conditions, the pure water flux of the membrane could reach 464 LMH, and the pore size distribution was highly concentrated at 72 nm. In addition, we assembled a membrane module and tested its practical application by filtering the reservoir water, proving the membrane’s application potential.

## 2. Materials and Methods

### 2.1. Chemicals

The main polymer, polyethersulfone (PES), was purchased from BASF (Ludwigshafen, Germany) with a mean average molecular weight (MW) of 58 kDa. N,N-dimethylacetamide (DMAc, >99%), citric acid (>99%), and triethylene glycol were purchased from Shanghai Macklin Biochemical Technology Co., Ltd., Shanghai, China.

Carboxylic graphene quantum dots (CGQDs) were synthesized through the pyrolysis of citric acid according to the previous study [18,19]. The synthesis process is as follows: citric acid (50 g) was added to a crucible (250 mL), heated at 200 °C until the solid melted completely, kept at 200 °C for 50 min, and then cooled to room temperature; a brown viscous liquid was obtained.

### 2.2. Preparation of Seven-Channel PES Hollow-Fiber Membrane

**Figure 1:**
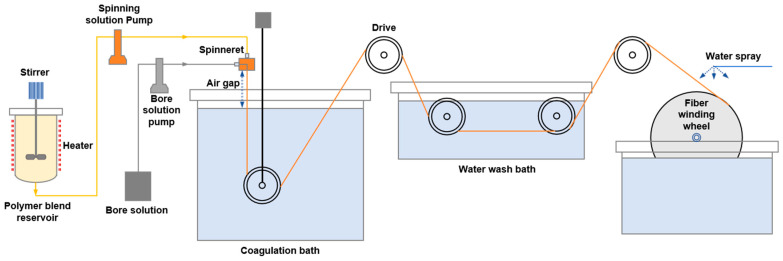
PES hollow-fiber spinning apparatus.

A seven-channel PES hollow-fiber membrane was prepared with the vapor-induced phase separation (VIPS) method. As shown in Figure 1, polymers (PES, CGQDs, and PEG) and solvents (Triethylene glycol, DMAc) were mixed in the polymer blend reservoir and stirred for 12 h at 80 °C. After the polymers had completely dissolved, the mixed solution was cooled to 40 °C and vacuum-degassed at −0.08 MPa for 12 h. The polymer solution, spinneret, bore solution, and coagulation bath solution were heated to the target temperature, and then the polymer solution and bore solution were pumped into the special spinneret using gear pumps. The appearance and design parameters of the spinneret are shown in Figure S1 (Supporting Information). After passing through an air gap, the polymer solution entered the coagulation bath, passed through the guide wheel, and entered the water washing tank, and was finally rolled up to obtain the hollow-fiber membrane. Table 1 lists the composition of the spinning solution. The mass ratio of PES, water, and TEG was determined through preliminary experiments and references [13,20]. Table 2 lists the composition of the bore solution. Table 3 lists the hollow-fiber membrane preparation process conditions.

### 2.3. Characterization

#### 2.3.1. Membrane Morphology and Structure

The surface and cross-sectional morphology of the membrane were tested using a scanning electron microscope (SEM, TM4000 Plus II, HITACHI, Tokyo, Japan; and Sigma 360, Zeiss, Oberkochen, Germany). The membrane sample was first dried at 40 °C and cut into small pieces and then pasted on the sample table with conductive adhesive. For the cross-sectional membrane samples, the membrane was first immersed in liquid nitrogen and then broken after the membrane became fragile, and the membrane was pasted on the sample table with conductive adhesive. Due to the poor conductivity of polymers, gold was sprayed on for 40 s using a vacuum-coating machine to improve the membrane’s conductivity.

#### 2.3.2. Infrared Spectral Analysis

In this study, the incorporation of additives into polymer membranes was detected using FT-IR spectroscopy (ALPHA II; Bruker, Billerica, MA, USA). With wavelengths spanning from 550 to 4000 cm^−1^, transmission tests were conducted on a membrane sample of the proper size that was placed on a sample holder.

#### 2.3.3. Surface Contact Angle and Zeta Potential Test

The contact angle of pure water on the membrane surface was determined using a contact angle meter (OCA25; DataPhysics Instruments, Filderstadt, Germany). The dried membranes were cut to a suitable size, adhered to a clean glass slide, and pasted onto the sample stage. At room temperature, a microsyringe was used to drop 3 μL of ultrapure water onto the sample surface, and then the contact angle between the water droplet and the sample was tested with the assistance of software. A zeta potential analyzer and a surface zeta potential electrode (NanoBrook-Omni, Brookhaven, Nashua, NH, USA) were used to measure the surface potentials of membranes using a solution of 0.025 mg/mL BI-ZR5 and 1 mM KCl at 25 °C and pH = 7. The relationship between zeta potential and distances was established by measuring the zeta potentials of the standard solution at various known distances from the membrane surface. The standard curve was then used to determine the membrane surfaces’ zeta potentials.

#### 2.3.4. Membrane Pore Size Distribution Analysis

This study used the BSD-PB series Full Function Membrane Pore Size Analyzer (BSD Instrument Technology (Beijing) Co., Beijing, China) with the liquid–liquid porosimetry method to analyze the membrane pore size distribution. After infiltrating the membrane to be tested with a completely saturated solution, a liquid that was not miscible with the infiltration solution was used as the displacement fluid. Then, the infiltration solution was displaced out of the through-hole channel to obtain the liquid flow rate and pressure data. According to the Washburn formula, the pore diameter data of the filter material was obtained. Due to the much lower interfacial tension between liquid and liquid compared to the gas–liquid displacement method (bubble pressure method), the liquid–liquid displacement method can test filter materials with smaller pore sizes. In this study, *n*-Butanol was used as a displacement fluid.

The relationship between pore size and pressure is shown in the Washburn formula [21]:

(1) $$D=\frac{4\gamma Cos\theta }{p}$$

where *D* is the pore diameter; *γ* is the surface tension of liquids; *θ* is the contact angle; and *p* is the differential pressure.

The flow percentage of pore size distribution is as follows:

(2) $$f\left(D\right)=\frac{-d\left(F_{w}-F_{d}\right)\times 100}{dD}$$

where *F_w_* = wet sample flow rate; *F_d_* = dry sample flow rate.

#### 2.3.5. Membrane Mechanical Performance Test

The mechanical properties of the membrane were tested using a digital tensile machine (UTM5000 series, Shenzhen Suns Technology Stock Co., Ltd., Shenzhen, China). The membrane was cut into 15 cm lengths and fixed on the clamp of the tensile machine. The membrane was stretched at a speed of 0.2 mm/s until the membrane broke, and the maximum tensile force was recorded.

### 2.4. Membrane Performance Test

#### 2.4.1. Pure Water Flux Tests

A homemade device tested the membrane’s pure water flux. The hollow fiber was encapsulated in a plastic tube with epoxy resin glue, and the two ends were cut off to expose the inner surface of the membrane. The membrane pure water flux was tested using the internal pressure method. The distilled water pressure was compressed to 0.4 bar using a peristaltic pump, and the volume of water *V* that passed through within the test time *t* was tested. The membrane pure water flux can be calculated using Formula (3).

(3) $$J=\frac{V}{7\pi d\times L\times P\times t}$$

where *J* represents the pure water flux (L · m^−2^ · h^−1^ · bar^−1^), *V* is the volume of water passing through the membrane in the time period *t*, *d* is the inner channel diameter of the fiber, *L* is the effective length of the fiber, and *P* is the test pressure (0.4 bar).

#### 2.4.2. Membrane Filtration Performance Tests

PBS buffer solution (0.01 M) was used to prepare a 1 g/L BSA solution for the membrane filtration performance test. The membrane BSA rejection rate was then tested using the pure water flux test device. The BSA concentration was tested using a membrane UV spectrophotometer (L5 series, INESA (Group) Co., Ltd., Shanghai, China).

#### 2.4.3. Membrane Long-Term Filtration Performance Test

In order to verify the water purification efficiency of the membrane prepared in this study, we conducted long-term tests on the membrane and examined the changes in membrane flux, inlet and outlet water turbidity, and transmembrane pressure difference over time. In the membrane filtration system, the membrane flow rate was set at 50 L · m^−2^ · h^−1^, and the water entering the membrane came from a reservoir that had been treated with coagulation and sedimentation. The membrane was backwashed for 30 s every 24 min of operation, and the backwash flow was set at 150 L · m^−2^ · h^−1^.

#### 2.4.4. Water Quality Parameter Test

The turbidity of water was tested using the MIK-PTU300 online turbidimeter (Hangzhou Meacon Co., Ltd., Hangzhou, China). The TOC of water was tested using the Wet Oxidation TOC Analyzer (TOC-V CPH 200V, Shimadzu Corporation, Kyoto, Japan).

## 3. Results and Discussion

### 3.1. Influence of CGQD Mass Fraction on Membrane Microstructure and Performance

#### 3.1.1. Microstructure of Membranes

**Figure 2:**
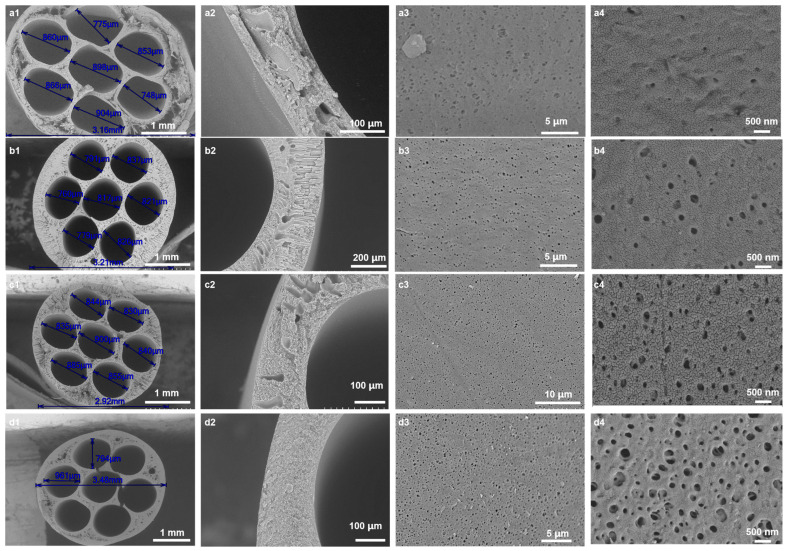
Cross-section and outlet surface SEM images of the PES membrane prepared with different CGQD mass fractions: (**a1**–**a4**) 0 wt%; (**b1**–**b4**) 3 wt%; (**c1**–**c4**) 6 wt%; (**d1**–**d4**) 7.5 wt%.

Figure 2 shows SEM images of the cross-section and external surface of the hollow-fiber membranes prepared under different conditions. As shown in Figure 2, all membranes have an asymmetric structure, with a dense separation layer on the surface and a finger-like or sponge-like structure inside. When the mass fraction of CGQD is 0 wt%, due to the low viscosity of the spinning solution, the instantaneous phase inversion leads to the stratification of the inside and outside of the membrane (Figure 2(a1,a2)). As the mass fraction of CGQD increases from 0% to 3%, the internal stratification of the membrane disappears; finger-like pores appear inside the membrane, and the surface porosity increases (Figure 2(b1,b2)). As the mass fraction of CGQD increases from 3% to 7.5%, the number of finger-like pores in the membrane decreases, and the membrane’s surface porosity is further improved (Figure 2(c1–c3,d1–d3)). The number of large and finger-like pores in the membrane is reduced due to the slower phase inversion, which occurs because the spinning solution becomes thicker with a higher CGQD mass fraction. However, if the mass fraction of CGQD exceeds 7.5%, then the casting solution will exceed the turbid point (Figure S2, Supporting Information). Therefore, the maximum mass ratio of CGQDs was set to 7.5%.

#### 3.1.2. Chemical Composition, Water Contact Angle, and Zeta Potential

**Figure 3:**
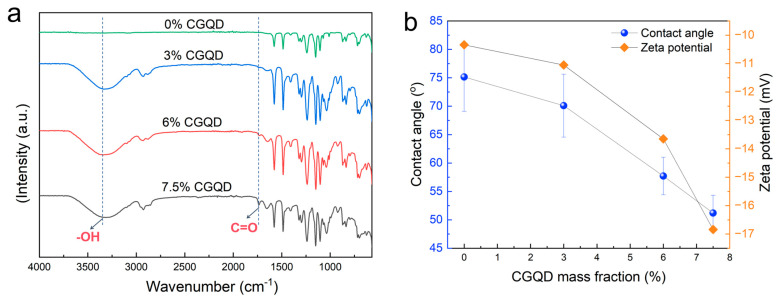
(**a**) FTIR spectrum and (**b**) contact angle and surface Zeta potential of PES membrane prepared with different CGQD mass fractions.

The chemical composition of PES membranes was analyzed by FTIR (Figure 3a). All PES membranes with added CGQDs show a C=O stretching vibration peak at about 1730 cm^−1^, and the peak intensity increases with the increase in CGQD addition [18]. In contrast, the membranes without added CGQDs do not show C=O stretching vibration peaks at 1730 cm^−1^ or O-H vibration stretching peaks at 3000–3600 cm^−1^, indicating that a large amount of the CGQDs added remain inside the membrane. Since CGQD contains a large number of carboxyl groups, the CGQDs retained inside the membrane could help improve its hydrophilicity. As shown in Figure 3b, the contact angle of the membrane surface decreases from 75.1° to 51.2° as the amount of CGQD added increases, and the membrane surface Zeta potential decreases from −10.34 mV to −16.84 mV as the amount of CGQD added increases. The decreases in water contact angle and Zeta potential indicate that the hydrophilicity of the membrane surface has been significantly improved.

#### 3.1.3. Pure Water Flux, BSA Rejection, and Mechanical Properties

**Figure 4:**
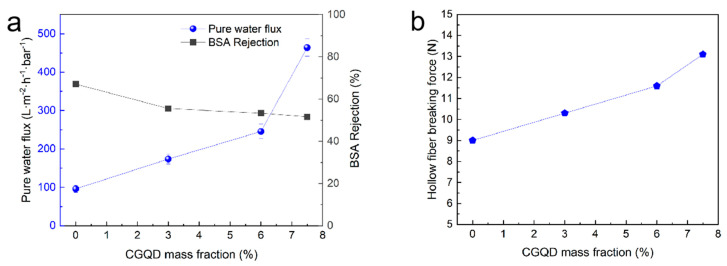
(**a**) Pure water flux, BSA rejection rate of PES membrane prepared with different CGQD mass fractions; (**b**) hollow-fiber breaking force (maximum load) of PES membrane prepared with different CGQD mass fractions.

Figure 4a shows the pure water flux and BSA rejection rate of membranes prepared under different conditions. With the increase in CGQD content, the pure water flux of the membrane gradually increased, and the BSA rejection rate slowly decreased. This phenomenon can be attributed to the increase in the porosity of the membrane surface, as shown in Figure 2. When the mass fraction of CGQD is 7.5%, the membrane pure water flux reaches 464 L·m^−2^ · h^−1^ · bar^−1^, and the BSA rejection rate reaches 51.6%. The breaking force is the maximum load that the hollow-fiber membrane can withstand before being broken, which can reflect the membrane’s ability to resist impact and vibration. As shown in Figure 4b, the breaking force of the membrane increases from 9 N to 13.1 N with the increased mass fraction of CGQD. The improvement in the mechanical properties of the membrane can be attributed to the reduction in the number of macrovoids and finger-like pores inside the membrane.

### 3.2. Influence of Air Gap and Relative Humidity on Membrane Microstructure

**Figure 5:**
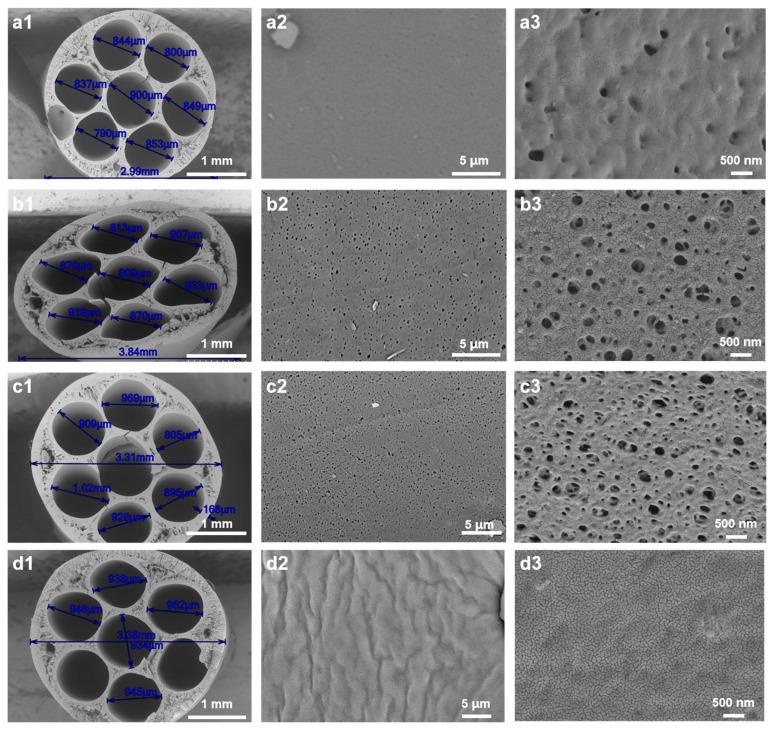
Cross-section and outlet surface SEM images of the PES membrane prepared with different air gap and relative humidity conditions: (**a1**–**a3**) 7.5 cm air gap and 50% RH; (**b1**–**b3**) 7.5 cm air gap and 99% RH; (**c1**–**c3**) 10 cm air gap and 99% RH; (**d1**–**d3**) 15 cm air gap and 99% RH.

In the hollow-fiber membrane preparation process, the distance between the spinneret and the coagulation bath is called the air gap [13]. Various mass transfer phenomena may occur before the nascent fiber reaches the non-solvent [15,22,23]. The length of the air gap and the relative air humidity are particularly important for controlling the membrane morphology. Figure 5 shows the hollow-fiber membranes prepared with various humidity conditions and air gaps (Table 4). The membranes in Figure 5a were prepared with a 10 cm air gap and 50% humidity, while the membranes in Figure 5b–d were prepared with 99% humidity and air gaps of 7.5, 10, and 12.5 cm, respectively. As can be seen from Figure 5(a2,a3), no obvious pores can be observed on the membrane surface under the condition of 50% relative humidity; when the relative humidity is increased to 99%, the membrane surface porosity increases significantly (Figure 5(b2,b3)). At 99% humidity, the casting solution can absorb water from the air and undergo phase transformation, but insufficient water absorption can only lead to phase separation on the membrane surface. Phase separation on the membrane surface could inhibit the rapid exchange of solvent and non-solvent in the coagulation bath and inhibit the formation of finger-like pores and macrovoids. At the same time, the microdroplets of water in the nearly saturated air could act as the nucleus of phase transformation, and this partial phase separation caused by water microdroplets could increase the porosity of the membrane surface. Further increasing the air gap could increase the time for which the polymer solution was in contact with humid air. As can be seen in Figure 5(c2,c3), when the air gap increases from 7.5 cm to 10 cm, the pore density of the membrane surface increases, which is consistent with previous reports [15]. However, when the air gap was increased from 10 cm to 15 cm, the membrane surface underwent a more complete phase transformation and formed a dense surface due to the longer exposure time to humid air (Figure 5(d2,d3)). Therefore, in this study, a 10 cm air gap and 99% relative humidity were used as the optimal conditions for membrane preparation.

### 3.3. Membrane Pore Size and Pore Distribution

**Figure 6:**
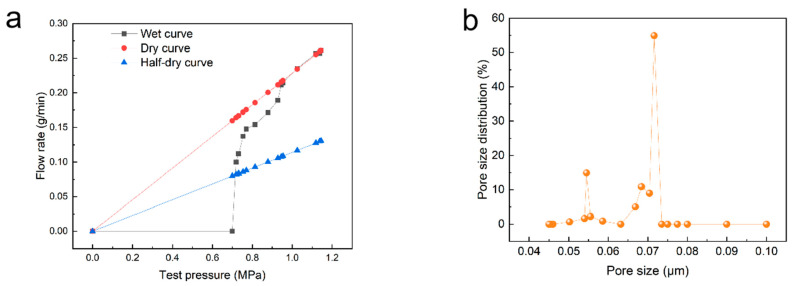
(**a**) The flow–pressure curve and (**b**) pore diameter distribution of the PES membrane prepared under optimal conditions.

In order to analyze the separation accuracy of the membrane prepared under the optimized conditions, the pore size distribution of the membrane was tested using the bubble point method. Because the membranes made in this study had pore sizes smaller than 100 nm, the usual gas–liquid bubble pressure method needed a test pressure higher than 0.3 MPa, which may have broken the hollow-fiber membrane. Therefore, this study used the liquid–liquid porosimetry method to test the membrane pore size distribution. Figure 6a shows the membrane flow–pressure curve. The membrane flow rate undergoes a sudden change at 0.7 bar. The pore size distribution of the membrane calculated by the Washburn formula is shown in Figure 6b. It can be seen that 54.9% of the pore size distribution is 0.072 microns, indicating that the membrane pore size distribution has a high concentration and is expected to accurately intercept large-molecular pollutants in water.

### 3.4. Membrane Long-Term Filtration Performance

**Figure 7:**
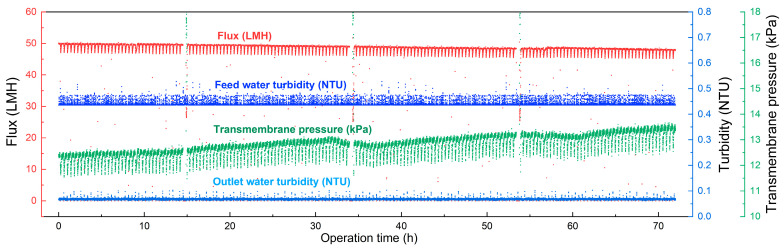
The flux, feed and outlet water turbidity, and transmembrane pressure difference over time in the long-term membrane filtration test.

In order to verify the water purification efficiency of the membrane prepared in this study, we conducted long-term tests on the membrane and examined the changes in membrane flux, inlet and outlet water turbidity, and transmembrane pressure difference over time. In the membrane filtration system, the membrane flow rate was set at 50 L·m^−2^ · h^−1^, and the water entering the membrane came from a reservoir that had been treated with coagulation and sedimentation. The membrane was backwashed for 30 s every 24 min of operation, and the backwash flow was set at 150 L·m^−2^ · h^−1^. It can be seen from Figure 7 that the turbidity of the membrane outlet water was stable at 0.066 NTU, and the removal rate of turbidity was about 85%. We also tested the removal rate of TOC. As shown in Table 5, the membrane prepared in this study can reject more than about 15% of TOC. In the filtration performance test, the transmembrane pressure of the membrane increased about 0.3–0.6 kPa per day; based on this growth rate, the clean-in-place (CIP) chemical cleaning period can exceed 30 days, which shows that the membrane prepared in this study has great potential in water purification applications.

## 4. Conclusions

In this study, CGQD additives were synthesized and used in the preparation of a seven-channel hollow-fiber membrane. CGQD is a type of polymerized organic acid that can act as a pore-former in membrane structure regulation, and the carboxyl groups it carries can also enhance its hydrophilicity and anti-fouling performance. As the amount of CGQD in the casting solution increased from 0 to 7.5, the membrane surface porosity increased, the number of macrovoids and finger-like pores inside the membrane decreased, and the membrane flux improved from 96 to 464 L·m^−2^ · h^−1^ · bar^−1^. This study found that the relative humidity and length of the air gap in VIPS could influence the membrane surface porosity and membrane morphology. Increasing the relative humidity of the air gap from 50% to 99% could increase the membrane surface porosity and reduce the number of macrovoids inside the membrane. The possible reason is that the partial phase separation on the membrane surface caused by humid air reduced the rapid phase separation of the casting solution, which could avoid the formation of macrovoids and increase the membrane surface porosity. The pore size of the membrane prepared under optimal conditions was tested by the liquid–liquid porosimetry method, and the pore size distribution of the membrane was concentrated at 72 nm. In the long-term filtration test, the turbidity of the filtered water was as low as 0.066 NTU, and the TOC removal rate of the membrane exceeded 15%. The seven-channel membrane prepared in this study is expected to be used in applications such as municipal water treatment, industrial recycled water treatment, and seawater desalination pre-treatment.

## Figures and Tables

**Table 1:** Composition of the spinning solution.

ID	C_PES_	C_CGQDs_	C_water_	C_TEG_	C_DMAc_
	wt%				
a	18	0	1.5	20	60.5
b	18	3	1.5	20	57.5
c	18	6	1.5	20	54.5
d	18	7.5	1.5	20	53

**Table 2:** Composition of the bore solution.

C_water_ (wt%)	C_DMAc_ (wt%)	C_TEG_ (wt%)
55	20	25

**Table 3:** Hollow-fiber membrane preparation process conditions.

Process Conditions	Parameters
Feed tank operating temperature (°C)	40
Bore solution tank operating temperature (°C)	25
Gear pump operating temperature (°C)	40
Spinning solution Flux (mL/min)	12
Bore solution Flux (mL/min)	35
Coagulation bath	Tap water
Temperature of the coagulation bath (°C)	25
Air gap (cm)	7.5–15
Air gap temperature (°C)	25
Air gap relative humidity (%)	50–99%

**Table 4:** Air gap and relative humidity of hollow-fiber membrane preparation process.

ID	Air Gap (cm)	Relative Humidity (%)	Air Exposure Time (s)
Figure 5(a1–a3)	7.5	50	0.75
Figure 5(b1–b3)	7.5	99	0.75
Figure 5(c1–c3)	10	99	1
Figure 5(d1–d3)	15	99	1.5

**Table 5:** Feed and outlet water TOC, TOC rejection rate, and transmembrane pressure increase rate in the long-term membrane filtration test.

Operation Time	Feed Water TOC (mg/L)	Outlet Water TOC (mg/L)	TOC Rejection Rate (%)	Transmembrane Pressure Increase (kPa)
Day1	1.12	0.92	17.8	0.617
Day2	1.19	1.01	15.1	0.315
Day3	1.30	1.02	21.6	0.389

## Data Availability

Data are contained within the article.

## Supplementary Materials

The following supporting information can be downloaded at: https://www.mdpi.com/article/10.3390/membranes15060175/s1, Figure S1: Appearance and design parameters of seven channel spinneret; Figure S2: Digital photos of spinning solutions with different CGQD addition amounts.
