# Supplementary material for: Seven-Channel Polyethersulfone Hollow-Fiber Membrane Preparation with Vapor-Induced Phase Separation
Source: Membranes (Basel). 2025 Jun 10;15(6):175. doi: 10.3390/membranes15060175 (PMC12194855; doi:10.3390/membranes15060175)
Supplement: Supplementary file 1 [file membranes-15-00175-s001.zip › membranes-3637447-supplementary.pdf]

Supplementary Materials

Seven-Channel Polyethersulfone Hollow-Fiber Membrane Preparation with Vapor-Induced Phase Separation

Xiaoyao Wang [1,2,*,†], Zhiyuan Hao [3,†], Rui Huang[1], Yajing Huang[1], Huiqun Zhang[1] and Xiujuan Hao[4,*]

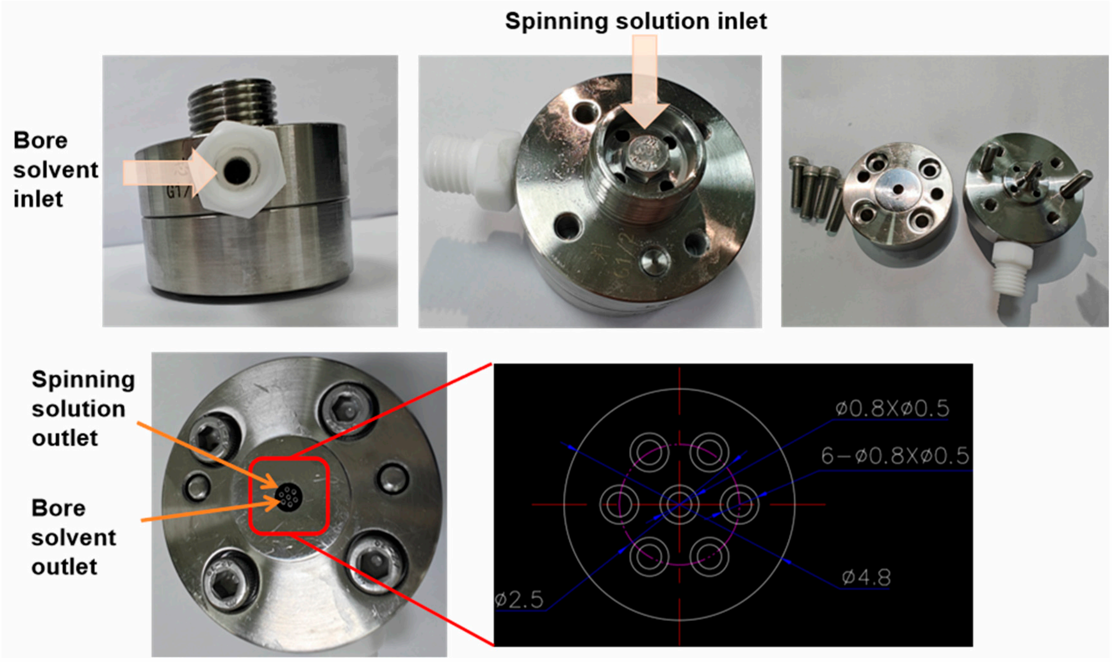

Figure S1. Appearance and design parameters of seven channel spinneret.

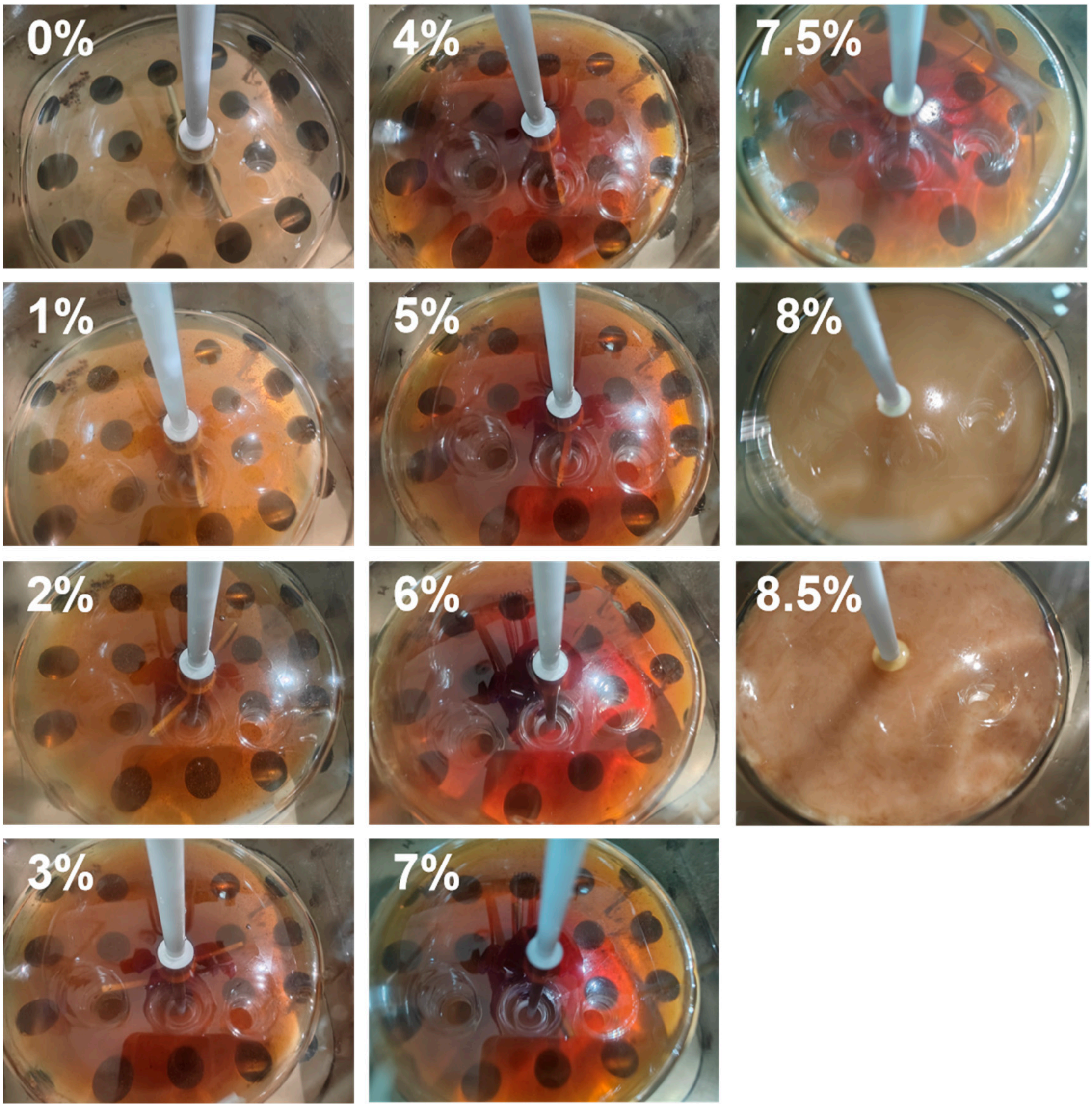

Figure S2. Digital photos of spinning solutions with different CGQD addition amounts.
